# Comparative analysis of background EEG activity based on MRI findings in neonatal hypoxic-ischemic encephalopathy: a standardized, low-resolution, brain electromagnetic tomography (sLORETA) study

**DOI:** 10.1186/s12883-022-02736-9

**Published:** 2022-06-03

**Authors:** Kwang Yeon Kim, Joo-Young Lee, Ja-Un Moon, Tae-Hoon Eom, Young-Hoon Kim

**Affiliations:** Department of Pediatrics, College of Medicine, The Catholic University of Korea, Seoul, Republic of Korea

**Keywords:** Hypoxic-ischemic encephalopathy (HIE), Quantitative electroencephalography (qEEG), Distributed source model, Standardized low-resolution brain electromagnetic tomography (sLORETA)

## Abstract

**Background:**

It is important to assess the degree of brain injury and predict long-term outcomes in neonates diagnosed with hypoxic-ischemic encephalopathy (HIE). However, routine studies, including magnetic resonance imaging (MRI) and conventional encephalography (EEG) or amplitude-integrated EEG (aEEG), have their own limitations in terms of availability and accuracy of evaluation. Recently, quantitative EEG (qEEG) has been shown to improve the predictive reliability of neonatal HIE and has been further refined with brain mapping techniques.

**Methods:**

We investigated background EEG activities in 29 neonates with HIE who experienced therapeutic hypothermia, via qEEG using a distributed source model. MRI images were evaluated and classified into two groups (normal-to-mild injury *vs* moderate-to-severe injury), based on a scoring system. Non-parametric statistical analysis using standardized low-resolution brain electromagnetic tomography was performed to compare the current density distribution of four frequency bands (delta, theta, alpha, and beta) between the two groups.

**Results:**

Electrical neuronal activities were significantly lower in the moderate-to-severe injury group compared with the normal-to-mild injury group. Background EEG activities in moderate-to-severe HIE were most significantly reduced in the temporal and parietal lobes. Quantitative EEG also revealed a decrease in background activity at all frequency bands, with a maximum in decrease in the delta component. The maximum difference in current density was found in the inferior parietal lobule of the right parietal lobe for the delta frequency band.

**Conclusions:**

Our study demonstrated quantitative and topographical changes in EEG in moderate-to-severe neonatal HIE. They also suggest possible implementation and evaluation of conventional EEG and aEEG in neonatal HIE. The findings have implications as biomarkers in the assessment of neonatal HIE.

## Background

Hypoxic-ischemic encephalopathy (HIE) is the leading cause of death in newborns and is also associated with long-term neurodevelopmental disabilities. HIE occurs in 1–2/1000 live births and accounts for 23% of neonatal deaths [1, 2]. Therapeutic hypothermia (TH) has now become the standard of care for near-term neonates with moderate-to-severe HIE and has been shown to improve survival and neurodevelopmental outcome [3–6]. In addition, assessment of cerebral injury and classification of severity in HIE are important for long-term prognosis and treatment planning. Many diagnostic modalities, including encephalography (EEG) and magnetic resonance imaging (MRI) have been effective in HIE. The amplitude-integrated EEG (aEEG) is now widely used with the introduction of TH as a bedside tool. These modalities play an important role in prognosis [1, 7]. Nevertheless, these routine studies have their own limitations. Although MRI is the most sensitive imaging test, it is often challenging for critically ill patients due to transportation limitations and the need for sedation [8]. The aEEG is a useful test for neonatologists; however, conventional EEG is still needed for precise evaluation [7].

Some recent studies using quantitative EEG (qEEG) have reported improved predictive reliability in neonatal HIE [9–11]. Quantitative EEG analysis is an excellent tool to evaluate background EEG and utilizes advanced brain-mapping techniques [12]. During brain mapping, the distributed source model is advantageous, and its algorithms address the inverse problem with few lead-in assumptions [13, 14]. This model represents a sophisticated method for investigating the electrophysiological and anatomical distributions of the brain. However, none of the studies investigated the EEG characteristics of neonatal HIE using a distributed model. The distributed source model might be a useful approach to assess the electrophysiological properties and cerebral injury in neonatal HIE. Thus, we performed a comparative qEEG analysis of background activity in neonatal HIE, based on MRI findings utilizing a distributed source model.

## Methods

### Patients and cooling protocol

A total of 29 neonates (17 males, 12 females) with HIE, who underwent TH and also had multi-channel EEG and brain MRI at our hospital from September 2016 to August 2021, were enrolled in this study*.* The study was approved by the Institutional Review Board and the parents of all neonates provided written informed consent prior to data acquisition.

TH with whole-body cooling (Blanketrol® III Hyper-Hypothermia System, Cincinnati Sub-Zero; Cincinnati, OH, USA) was administered within 6 h of birth, according to our institutional guidelines as follows: 1) gestational age ≥ 35 weeks; 2) birth weight ≥ 2,000 g; 3) presence of ≥ 1 of the following parameters: pH < 7.0 or base deficit > 6 mmol/L on cord or first blood gas, or Apgar score ≤ 5 at 10 min, or ventilatory support (intubation or mask ventilation) needed at birth and continued at 10 min of life; 4) the presence of moderate-to-severe encephalopathy identified by modified Sarnat Staging System. Exclusion criteria for TH included coagulopathy with active bleeding and known or suspected congenital anomalies or metabolic disorders. A target temperature of 33.5 ± 0.5 °C was maintained for 72 h, from the time core rectal temperature was achieved, followed by gradual rewarming over 6 h. Sedation was performed for all neonates using a continuous infusion of fentanyl all over cooling to prevent any abnormal movement. During TH, the aEEG monitor was used as a bedside tool according to our clinical protocol. After rewarming, all neonates underwent additional conventional multi-channel EEG for detailed evaluation within 1 week. Brain MRI was also performed at least within 10 days of birth after rewarming and extubation due to changes in MR diffusion disappearing after the first week of life.

### Multi-channel EEG recordings and data processing

The details of the EEG recording were the same as those used in previous our study [15, 16]. Conventional multi-channel EEG recordings were performed for 3 h using a Comet® EEG machine (Grass-Telefactor; West Warwick, RI, USA) at a sampling rate of 200 Hz. Thirteen Ag/AgCl electrodes were placed according to the international 10–20 system adapted for neonates, including eight scalp electrodes (FP2, C4, T4, O2, FP1, C3, T3, and O1) together with Fz, Cz, and Pz [17]. Additional electrodes were also employed, including recordings of respiration and electrocardiography. Eleven-channel EEG was recorded using an average reference. Additional bipolar montages were used to differentiate between EEG and eye movement potentials and detect electromyographic activity. Electrode impedance did not exceed 5 kΩ. In the EEG derivations, the filters were set at 1.0 and 70 Hz. Sixteen-bit online digitization was used.

Each EEG recording was transformed using the fast Fourier transform (FFT) technique on 3-s manually segmented, artifact-free epochs (at rest without non-stationary elements, such as epileptiform or paroxysmal discharges) in the background after visual inspection. This epoch length was adequate to compute an FFT and short enough to include a sufficient number of artifact-free segments. For each patient, a dataset of 40 epochs was collected for each examination. The segments were representative of the whole recording as they were selected randomly across the entire length of the EEG recording [15, 18]. The epochs were selected blindly by a single author and independently reviewed by a second author. The EEG recordings were exported into American Standard Code for Information Interchange (ASCII) files and imported into standardized low-resolution brain electromagnetic tomography (sLORETA) software.

### MRI recordings and classification

Brain MRI (3 Tesla) was acquired, including T1- and T2-weighted images, Fluid Attenuated Inversion Recovery (FLAIR), diffusion-weighted imaging (DWI), and apparent diffusion coefficient (ADC) sequences. MRI images were evaluated and classified into normal-to-mild and moderate-to-severe injuries based on a previously published scoring system by Barkovich et al. [19]. Normal-to-mild injuries were represented by a basal ganglia/thalamus score < 2 and a watershed score < 3. Basal ganglia/thalamus scores ≥ 2 or watershed pattern ≥ 3 suggest moderate-to-severe injuries.

### EEG analysis using sLORETA and statistical analysis

Demographic data of the two groups (moderate-to-severe injury *vs.* normal-to-mild injury) were compared and the variables were analyzed via Student’s t-test and Fisher’s exact test with a threshold of *p* < 0.05.

A comparative analysis of background EEG activity was performed via statistical non-parametric mapping (SnPM) of the two groups using sLORETA at four frequency bands (delta, 1.0–3.5 Hz; theta, 3.5–7.5 Hz; alpha, 7.5–12.5 Hz; and beta, 12.5–25.0 Hz). The limits of each frequency band were based on relevant prior studies [15, 16]. In the sLORETA images, the cortex is modeled as a collection of volume elements (6239 voxels, size 5 × 5 × 5 mm), restricted to the cortical grey matter, hippocampus, and amygdala in the digitized Montreal Neurological Institute (MNI) coordinates corrected to the Talairach coordinates [12, 20]. Scalp electrode coordinates on the MNI brain were based on the international 5% system [21].

The current density distributions in the two groups were compared via voxel-by-voxel analysis using the sLORETA software at the four frequency bands. Non-parametric statistical analyses (SnPM) of sLORETA images were performed for each contrast with built-in voxel-wise randomization tests (5000 permutations) and using a log-*F*-ratio statistic for dependent groups with thresholds of *p* < 0.01 and *p* < 0.05, corrected for multiple comparisons. Correction for multiple comparisons in SnPM with random permutations (5000 in the current study) has been shown to yield results similar to those obtained from statistical parametric mapping using a general linear model with multiple comparison corrections based on random field theory [22, 23].

The sLORETA algorithm resolves the inverse problem via distributed modeling method [12]. The EEG inverse problem suggests a limited number of electrodes from scalp [24]. sLORETA computes the current distribution in the full brain and generates three-dimensional images (6239 voxels, size 5 × 5 × 5 mm), with maximum similarities of orientation and strength between neighboring neuronal populations [12, 13]. The technique is not limited to a specific number or location of electrodes. Because the sLORETA self-corrects in multiple testing procedures involving all electrodes and/or voxels, and all time samples and/or discrete frequencies via random permutations (5000) in the current study, no further correction is required for multiple comparison [12, 22]. The sLORETA has proved to be an efficient tool for functional mapping because it is consistent with physiology and enables correct localization [12]. Additionally, the sLORETA localization has been independently validated [25, 26].

## Results

### Demographics and brain injury in MRI

The gestational ages of the 29 neonates ranged from 35.0 to 41.1 weeks with a mean of 39.1 ± 1.8 (standard deviation; SD). The birth weights ranged from 2.54 to 3.86 kg with a mean of 3.20 ± 0.38 (SD), and included 58.6% males and 41.4% females. Eleven neonates (37.9%) had moderate-to-severe injury and 18 (62.1%) showed normal-to-mild injury on MRI images. Demographic and clinical characteristics of subjects in the two subgroups (moderate-to-severe injury *vs.* normal-to-mild injury) are presented in Table 1, and there were no statistically significant differences.

**Table 1:** Demographic and clinical characteristics related to brain injury in MRI

Demographics and clinical characteristic	Moderate-to-severe MRI injury (*n* = 11)	Normal-to-mild MRI injury (*n* = 18)	*p*-value^a^
Gender (male/female)	7/4	10/8	0.72
Gestational age (weeks, mean ± SD)	38.5 ± 2.1	39.5 ± 1.5	0.17
Birth weight (kg, mean ± SD)	3.06 ± 0.36	3.28 ± 0.37	0.13
Cord or blood gas pH	7.19 ± 0.13	7.22 ± 0.12	0.49
Base deificit	10.2 ± 5.4	9.3 ± 1.1	0.7
Apgar at 1 min	4.5 ± 2.2	4.8 ± 2.0	0.37
Apgar at 5 min	5.8 ± 2.3	6.6 ± 1.9	0.32
Sarnat stage (II/III)	9/2	18/2	0.6

*SD* Standard deviation,

^a^Variables were analyzed by t-test and Fisher’s exact test with a threshold of *p* < 0.05

### EEG distributed source analysis using sLORETA

**Fig. 1:**
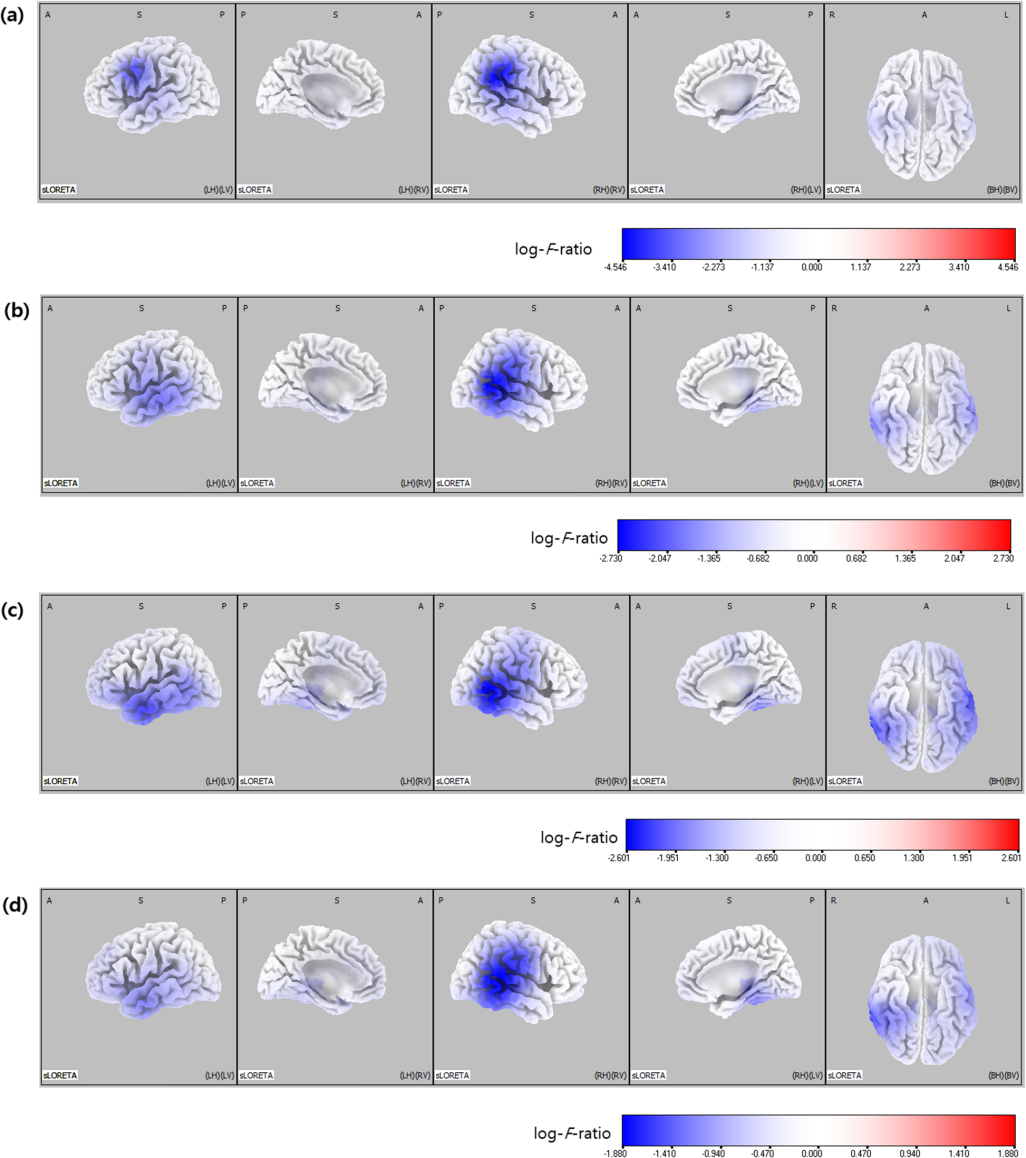
Statistical maps using standardized low-resolution brain electromagnetic tomography (sLORETA) of four frequency bands in the two groups (moderate-to-severe injury *vs.* normal-to-mild injury) were projected onto a three-dimensional fiducial brain cortex. Delta **(a)**. Theta **(b)**. Alpha **(c)**. Beta **(d)**. Non-parametric statistical analyses were performed to compare the current density distribution of the four frequency bands between the two groups. Log-*F*-ratio statistics were employed, and the color scale represents log-*F*-ratio values (threshold log-*F*-ratio =  ± 0.375, *p* < 0.05; threshold log-*F*-ratio =  ± 0.490, *p* < 0.01). A indicates anterior; *P* posterior, *S* superior, *I* inferior, *L* left, *R* right, *B* both, *H* hemisphere, *V* ventricle

Electrical neuronal activities at all four frequency bands were significantly lower in the moderate-to-severe injury group than in the normal-to-mild injury group (threshold log-*F*-ratio =  ± 0.375, *p* < 0.05; threshold log-*F*-ratio =  ± 0.490, *p* < 0.01). Comparative analysis using SnPM of the sLORETA data revealed significant differences in current density in all frequency bands throughout the cortex to a variable degree. Figure 1 shows the statistical maps of the spatial extent of the voxels within the areas of significant differences in current density in the three-dimensional fiducial cortical surface. Table 2 summarizes the location of the largest (top five) differences in current density.

**Table 2:** Locations of the largest (top five) differences in current density associated with background EEG activity in the two groups (moderate-to-severe MRI injury *vs.* normal-to-mild MRI injury)

	Hemisphere	Lobe	Log-*F*-ratio	*p*-value
*Delta frequency band*				
Inferior parietal lobule	Right	Parietal	-4.55	< 0.01
Insula	Right	Sub-lobar	-4.35	< 0.01
Supramarginal gyrus	Right	Temporal	-4.34	< 0.01
Superior temporal gyrus	Right	Temporal	-4.32	< 0.01
Postcentral gyrus	Right	Parietal	-4.29	< 0.01
*Theta frequency band*				
Superior temporal gyrus	Right	Temporal	-2.73	< 0.01
Middle temporal gyrus	Right	Temporal	-2.67	< 0.01
Inferior parietal lobule	Right	Parietal	-2.63	< 0.01
Insula	Right	Sub-lobar	-2.60	< 0.01
Supramarginal gyrus	Right	Temporal	-2.58	< 0.01
*Alpha frequency band*				
Middle temporal gyrus	Right	Temporal	-2.60	< 0.01
Superior temporal gyrus	Right	Temporal	-2.58	< 0.01
Inferior temporal gyrus	Right	Temporal	-2.45	< 0.01
Fusiform gyrus	Right	Temporal	-2.38	< 0.01
Inferior temporal gyrus	Left	Temporal	-2.38	< 0.01
*Beta frequency band*				
Superior temporal gyrus	Right	Temporal	-1.88	< 0.01
Middle temporal gyrus	Right	Temporal	-1.86	< 0.01
Postcentral gyrus	Right	Parietal	-1.81	< 0.01
Transverse temporal gyrus	Right	Temporal	-1.80	< 0.01
Insula	Right	Sub-lobar	-1.78	< 0.01

**Fig. 2:**
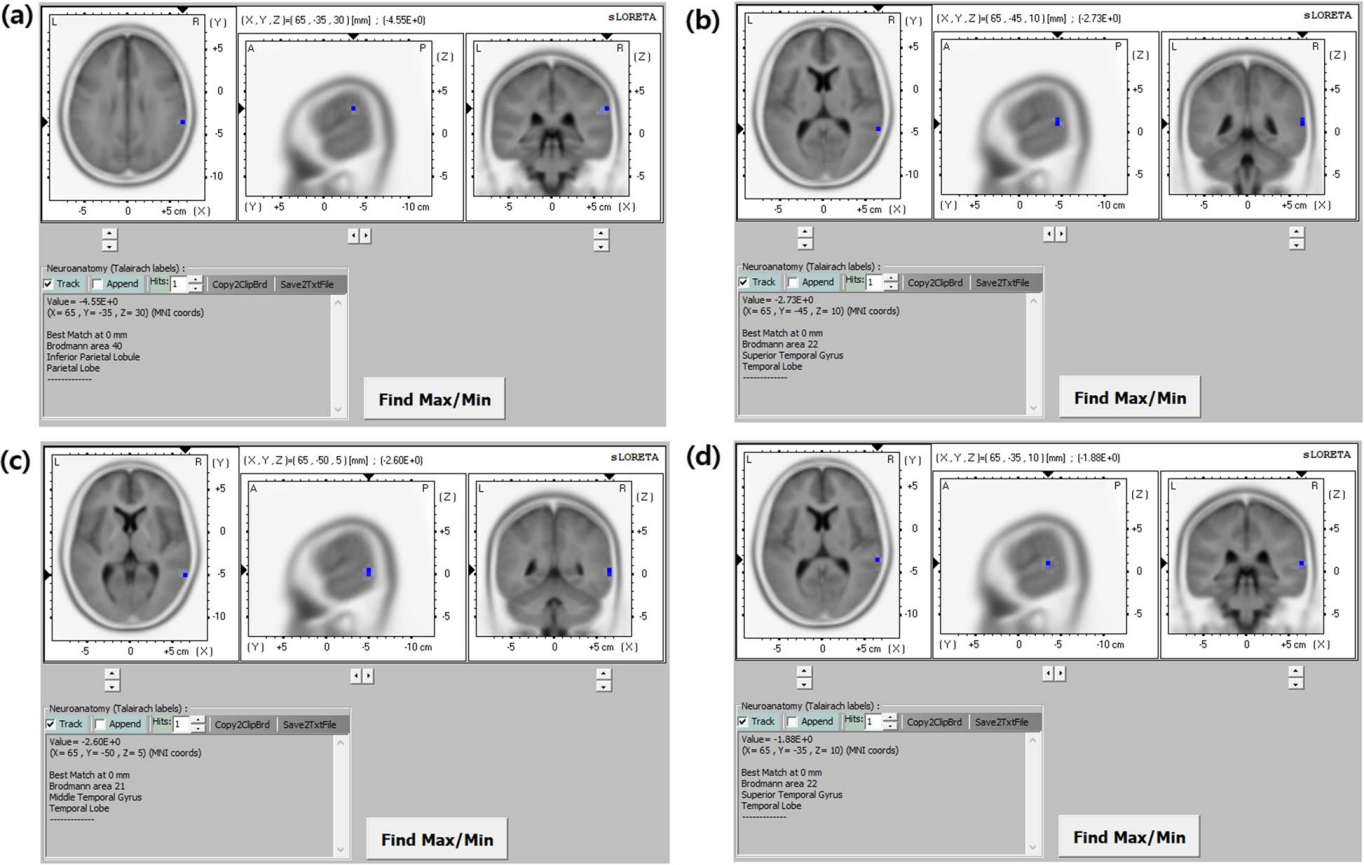
Statistical maps using standardized low-resolution brain electromagnetic tomography (sLORETA) of the four frequency bands in the two groups (moderate-to-severe injury *vs.* normal-to-mild injury) were projected onto a brain MRI template. Non-parametric statistical analyses were performed to compare the current density distributions of the four frequency bands between the two groups. Maximum current density differences were found in the right inferior parietal lobule for the delta frequency band (**a**), the right superior temporal gyrus for the theta frequency band (**b**), the right middle temporal gyrus for the alpha frequency band (**c**), and the right superior temporal gyrus for the beta frequency band **(d)**. MNI coord denotes Montreal Neurological Institute coordinate; *A* anterior, *P* posterior, *L* left, *R* right

The maximum difference in current density was found in the inferior parietal lobule of the right parietal lobe for the delta frequency band (MNI coordinate [x, y, z = 65, -35, 30], Brodmann area 40) (log-*F*-ratio =  − 4.55, *p* < 0.01) (Fig. 2a), the superior temporal gyrus of the right temporal lobe for the theta frequency band (MNI coordinate [x, y, z = 65, -45, 10], Brodmann area 22) (log-*F*-ratio =  − 2.73, *p* < 0.01) (Fig. 2b), the middle temporal gyrus of the right temporal lobe for the alpha frequency band (MNI coordinates x, y, z = 65, -50, 5, respectively; Brodmann area 21) (log-*F*-ratio =  − 2.60, *p* < 0.01) (Fig. 2c), and the superior temporal gyrus of the right temporal lobe for the beta frequency band (MNI coordinates x, y, z = 65, -35, 10, respectively; Brodmann area 22) (log-*F*-ratio =  − 1.88, *p* < 0.01) (Fig. 2d).

## Discussion

Currently, a variety of diagnostic modalities are available to assess the degree of brain injury and predict long-term outcomes in neonates with HIE [27]. In particular, the prognostic value of MRI and aEEG or conventional EEG has been well established [28]. Current MRI injury scoring systems have been shown to be consistent with neurodevelopmental outcomes [19, 29–31]. Also, aEEG or conventional multi-channel EEG performed within the first 7 days after birth in neonates with HIE is known to play a predictive role [28]. In addition, EEG background activities correlated well with MRI abnormalities [29].

However, these diagnostic tools have their own strengths and weaknesses, with unmet clinical needs. Although MRI is the most sensitive and specific imaging technique for hypoxic-ischemic brain injury, it is often challenging for critically ill patients due to transportation limitations and the need for sedation [8]. Conventional multi-channel EEG is the gold standard for neonatal HIE monitoring. However, recording and interpretation require specialized technicians and neurologists who are not readily available [32]. Given the simplicity of application and interpretation, aEEG has been widely used in intensive care settings; however, it cannot completely replace multi-channel EEG [33]. The aEEG technique is basically an EEG derived from qEEG techniques, and further sophisticated qEEG analysis using brain mapping techniques might have novel implications for HIE assessment [34].

Our study showed that background EEG activity in moderate-to-severe HIE was most significantly reduced in the temporal and parietal lobes. Quantitative EEG analysis also suggested a decrease in background activity at all frequency bands, with a maximum reduction in the delta component.

Our results suggest important implications for interpreting conventional multi-channel EEG in neonates with HIE, especially the background activity in the temporal and parietal lobes. In addition, our results indicate that changes of the delta frequency band in the background EEG are conspicuous in moderate-to-severe HIE. We speculated that these findings were related to neonatal EEG characteristics, along with vulnerable “watershed zone” in HIE [35, 36]. The parietal lobe is in close proximity to the vulnerable “watershed” regions in the border zones of arterial blood supply from all three cerebral arteries [36]. Further, our results revealed the maximum difference in current density in the inferior parietal lobule of the right parietal lobe for the delta frequency band. However, our results showed that in neonates with moderate-to-severe HIE, background EEG activities were particularly reduced in the temporal lobe and so-called centro-temporal region in addition to the parietal lobe. In addition, the neonatal EEG profile differs from that of the other age groups. The background activities in neonatal EEG involve low-frequency bands, mainly delta and theta [35]. These features of neonatal EEG are consistent with the frequency bands of current density in our results. Therefore, it is speculated that changes in background EEG activity in neonates with moderate-to-severe HIE are necessarily affected by these characteristics of neonatal EEG.

Our results also suggest another factor for implementing aEEG monitor in neonates with HIE. Currently, in most institutions, one-channel aEEG usually uses electrode positions P3 and P4, and two-channel aEEG uses electrode positions C3 and P3, C4 and P4. The use of new positions has not been evaluated in studies. Our study suggests that temporal electrodes (e.g., T3 and T4) in the aEEG may be facilitate the monitoring of background EEG activity in neonates with HIE. In addition, it might be helpful to monitor low-frequency bands in the background EEG, especially delta activity, suggesting the usefulness of techniques such as compressed spectral array (CSA) or envelope trends [34]. Recently, more sophisticated technologies such as multi-channel aEEG and CSA are increasingly being used for continuous EEG monitoring in intensive care units [37, 38]. These recent techniques also enable digital creation of display graphs containing transformed and compressed EEG recording data [30]. Our study also supports the need for these diagnostic modalities in managing neonates with HIE.

To our knowledge, this was the first study using qEEG analysis based on the distributed model in neonatal HIE. Our study demonstrated quantitative and topographical changes in EEG in moderate-to-severe neonatal HIE. These findings also have implications as biomarkers in the assessment of neonatal HIE. Furthermore, they also suggest clinical implications for evaluating neonatal HIE using aEEG and conventional EEG.

Nevertheless, this preliminary study has several limitations. Due to the retrospective design and clinical constraints, it was not possible to conduct each EEG and MRI on the same day consistently. Multichannel EEG in the early stages of therapeutic hypothermia, i.e., 24, 48 and 72 h after birth, was not included in this study. These factors affect the EEG and MRI patterns in the developing brain after injury. However, given the practical clinical constraints, our study parameters might be more applicable under clinical settings. Further prospective studies with a rigorous protocol are needed to elucidate these factors. In addition, the study used MRI as a short-term outcome measure and did not evaluate long-term neurodevelopment. Long-term follow-up is needed to corroborate our results based on further evidence supporting the association between neurophysiological parameters in qEEG and long-term neurodevelopment.

Lastly, the standard MNI brain used in the sLORETA software is not derived from the pediatric population, which differs from adults in head size, skull shape, and tissue conductivity [39]. However, head models of infants or neonates are not frequently used currently. Specific head models can provide highly accurate localization. Also, the high-density EEG with additional electrodes can improve the results of source reconstruction. However, the properties of sLORETA localization have been independently validated [25] with accuracy similar in high- and low-density EEG [40, 41]. Therefore, we believe that our results are justified.

## Conclusions

In conclusion, this study demonstrated that the background EEG activity was significantly decreased in moderate-to-severe HIE, particularly in the temporal and parietal lobes and delta frequency band. Furthermore, these findings suggest clinical implications for evaluating neonatal HIE using aEEG and conventional EEG. In addition, these findings have implications as markers of moderate-to-severe HIE.

We believe that our results contribute to further understanding of the electrophysiological characteristics in neonatal HIE. Until now, no study has reported background activities based on the distributed model in neonatal HIE. Extensive studies based on rigorous protocols, including extended EEG recordings and long-term neurodevelopmental follow-up are needed to further expand our understanding of neonatal HIE and corroborate our results.

## Data Availability

The datasets analyzed during the current study contain identifying information and are therefore unavailable publicly. Data can be made available through contacting the corresponding author.

## Abbreviations

HIE: Hypoxic-ischemic encephalopathy (HIE)
TH: Therapeutic hypothermia
EEG: Electroencephalography
MRI: Magnetic resonance imaging
aEEG: Amplitude-integrated encephalography
qEEG: Quantitative encephalography
FFT: Fourier transformation
sLORETA: Standardized low-resolution brain electromagnetic tomography
SnPM: Statistical non-parametric mapping
MNI coordinates: Montreal Neurological Institute coordinates
SD: Standard deviation
